# Antistress Action of Melanocortin Derivatives Associated with Correction of Gene Expression Patterns in the Hippocampus of Male Rats Following Acute Stress

**DOI:** 10.3390/ijms221810054

**Published:** 2021-09-17

**Authors:** Ivan B. Filippenkov, Vasily V. Stavchansky, Natalya Yu. Glazova, Elena A. Sebentsova, Julia A. Remizova, Liya V. Valieva, Natalia G. Levitskaya, Nikolai F. Myasoedov, Svetlana A. Limborska, Lyudmila V. Dergunova

**Affiliations:** 1 Institute of Molecular Genetics of National Research Center “Kurchatov Institute”, Kurchatov Sq. 2, 123182 Moscow, Russia; bacbac@yandex.ru (V.V.S.); utoshkautoshka@gmail.com (J.A.R.); lia97@mail.ru (L.V.V.); nglevitskaya@gmail.com (N.G.L.); nfm@img.msk.ru (N.F.M.); limbor@img.msk.ru (S.A.L.); lvd@img.msk.ru (L.V.D.); 2 Faculty of Biology, Lomonosov Moscow State University, Leninskie Gory, 119991 Moscow, Russia; tusy-g@yandex.ru (N.Y.G.); sebentsova@list.ru (E.A.S.)

**Keywords:** acute restraint stress, melanocortin peptides, anxiety-related behavior, RNA-Seq, gene expression

## Abstract

Natural melanocortins (MCs) have been used in the successful development of drugs with neuroprotective properties. Here, we studied the behavioral effects and molecular genetic mechanisms of two synthetic MC derivatives-ACTH(4–7)PGP (Semax) and ACTH(6–9)PGP under normal and acute restraint stress (ARS) conditions. Administration of Semax or ACTH(6–9)PGP (100 μg/kg) to rats 30 min before ARS attenuated ARS-induced behavioral alterations. Using high-throughput RNA sequencing (RNA-Seq), we identified 1359 differentially expressed genes (DEGs) in the hippocampus of vehicle-treated rats subjected to ARS, using a cutoff of >1.5 fold change and adjusted *p*-value (*Padj*) < 0.05, in samples collected 4.5 h after the ARS. Semax administration produced > 1500 DEGs, whereas ACTH(6–9)PGP administration led to <400 DEGs at 4.5 h after ARS. Nevertheless, ~250 overlapping DEGs were identified, and expression of these DEGs was changed unidirectionally by both peptides under ARS conditions. Modulation of the expression of genes associated with biogenesis, translation of RNA, DNA replication, and immune and nervous system function was produced by both peptides. Furthermore, both peptides upregulated the expression levels of many genes that displayed decreased expression after ARS, and vice versa, the MC peptides downregulated the expression levels of genes that were upregulated by ARS. Consequently, the antistress action of MC peptides may be associated with a correction of gene expression patterns that are disrupted during ARS.

## 1. Introduction

Growing evidence suggests that acute/chronic stress leads to altered body homeostasis, resulting in physiological changes linked to serious health risks [1]. Acute stress affects behavior, and learning and memory, in animals and humans [2]. Acute stressful events can provoke cognitive dysfunction, and mood and anxiety disorders [3]. The search for ways to provide pharmacological correction of negative stress consequences is an important problem for modern neurobiology research. Currently, when developing anti-stress drugs, great importance is attached to the creation of medicines based on natural regulatory proteins and peptides [4,5,6].

Melanocortins (MCs) are a group of biologically active, endogenous peptides that are expressed within the central nervous system and in several peripheral tissues, including the skin, stomach, kidney, intestine, immunocompetent myeloid, and lymphoid cells [7,8]. The MC system exerts major modulatory actions on homeostasis functions, regulating important physiological processes such as food intake, sexual behavior, pain sensitivity, stress responses, fever control, pigmentation, neuroprotection, and learning and memory [9,10]. All natural MC peptides are formed from the pro-opiomelanocortin precursor, encoding by the *Pomc* gene [11]. The MC family includes adrenocorticotropic hormone (ACTH), α-, β-, and γ-melanocyte-stimulating hormones (α-, β-, and γ-MSH) and shorter fragments. ACTH, α-, and β-MSH share the common sequence—Met–Glu–His–Phe–Arg–Trp, which corresponds to the ACTH(4–9) fragment and is the main determinant of their biological activity [12]. Based on extensive studies of the MCs, some new and enzyme-resistant analogues of these peptides were developed.

The MC derivatives, ACTH(4–7)PGP (Semax) and ACTH(6–9)PGP, contain a fragment of ACTH(4–7) (Met–Glu–His–Phe) and ACTH(6–9) (His–Phe–Arg–Trp), respectively. Both peptides contain the C-terminal tripeptide, Pro–Gly–Pro (PGP) to ensure resistance to peptidases. Semax and ACTH(6–9)PGP have been shown to exert nootropic and anxiolytic activity [13,14,15]. Furthermore, Semax administration attenuated cognitive impairment caused by acute stress [16] and diminished the effects of chronic unpredictable stress in Wistar rats [17]. The half-life of Semax in the body is several minutes; however, duration of the therapeutic effect of the peptide is up to 24 h [18]. The stability of ACTH(6–9)PGP and Semax is equivalent [19]. For both peptides, the ability to penetrate the blood-brain barrier and interact with brain structures has been established [20,21,22]. The polypeptide sequence of ACTH(6–9) is necessary and sufficient to activate most types of MC receptors [23,24,25,26,27]. At the same time, there are suggestions about the allosteric interaction of Semax and ACTH(6–9)PGP with a large number of different receptors [28,29]. However, the molecular mechanisms underlying its anti-stress effects remain unclear.

Recent studies indicate a significant contribution of the transcriptome to the regulation of recovery processes in the relief of stress conditions. An et al. (2019) performed the first profiling of mRNA and non-coding RNA in depressed patients who practiced the traditional Chinese physical therapy “Baduanjin” [30] and reported that the antidepressant effect of Baduanjin was associated with a genetic response, with the level of RNAs involved in immune function and inflammatory response pathways, and various signaling pathways, including IL-17 and TNF, significantly modulated. Moreover, these investigators elucidated the regulatory networks of mRNAs and non-coding RNAs associated with the antidepressant action of “Baduanjin” [30].

Selank peptide (Thr−Lys−Pro−Arg−Pro−Gly−Pro) has pronounced anxiolytic activity and acts as a stable neuropsychotropic, antidepressant, and anti-stress drug [31]. Moreover, Selank enhanced the effect of diazepam in reducing anxiety in unpredictable chronic mild stress conditions in rats [32]. Recently, an evaluation of the effect of administration of Selank peptide on the transcriptional profile of rat hippocampal cells was completed, using cDNA microarray technology [6], and revealed that dozens of genes significantly changed their expression levels. Among these genes, most related to protein products that included receptors, ion channels, and regulatory proteins involved in key functions such as learning and memory formation. Using PCR, it was shown that Selank affected the expression of several neurotransmission-related genes in the frontal cortex of rats, including the *Adcy7*, *Cx3cl1*, *Gabra6*, *Gabrb1*, *Gabrb3*, *Gabre*, *Gabrq*, *Hcrt*, *Slc6a1*, and *Slc6a11* genes [33]. These data suggest that Selank can modulate the gene expression profile of the GABAergic system, and neurosignaling and neuroreception.

We have shown that ARS procedure consisting of the combination of the restraint with bright light and intermittent noise led to cognitive impairment and behavioral alterations 30 min after stress termination [16]. Preliminary Semax administration attenuated cognitive impairment caused by this acute stress, but failed to effect behavioral consequences. The aim of the present work was to study the effects of two synthetic melanocortins on the behavioral consequences and transcriptomic changes induced by acute restraint stress. The time point post ARS (4–4.5 h) was chosen to study prolonged behavioral and transcriptional ARS effects. The hippocampus is a key brain structure involved in the modulation of the acute stress response and anxiety-related behavior [3]. In order to elucidate the molecular mechanisms underlying the effects of acute stress and the effects of two synthetic MC derivatives on the consequences of acute restraint stress (ARS), we assessed transcriptomic changes in the hippocampus of rats exposed to ARS.

These studies revealed normalizing effects of Semax and ACTH(6–9)PGP treatment on the emotional-related behavior of rats exposed to ARS. High-throughput RNA sequencing (RNA-Seq) allowed the identification of possible gene targets of the peptides, and suggested mechanisms associated with the peptide regulation of brain activity under normal and ARS conditions.

## 2. Results

### 2.1. Effects of MC Derivatives on Behavioral Responses to ARS

**Figure 1:**
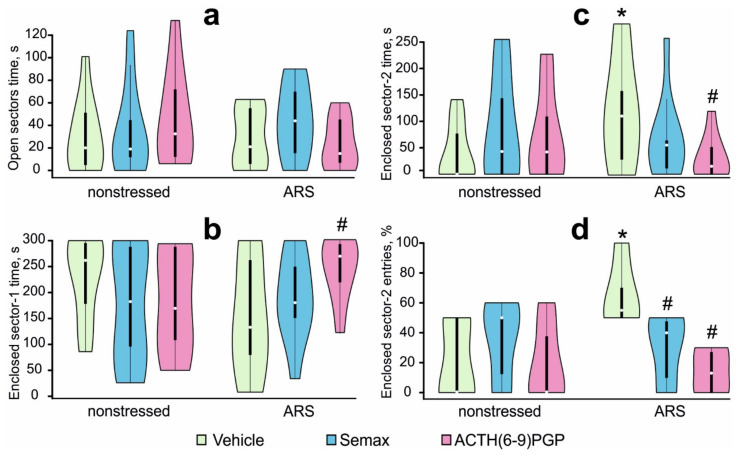
Effects of Semax and ACTH(6–9)PGP on rat behavior observed in the EZM 4 h after the termination of acute restraint stress (ARS). Time spent in the open sectors, s (**a**); time spent in the enclosed sector-1, s (**b**); time spent in the enclosed sector-2, s (**c**); percentage of entries in the enclosed sector-2, % (**d**) data are represented by Violin plots. Data are shown as mean ± SEM, *n* = 9–10 rats per group. Data were analyzed using two-way ANOVA followed by post hoc Dunnett’s test. Statistical difference is represented as * *p* < 0.05 versus NS-V group and # *p* < 0.05 versus STR-V group.

The effects of treatment with MC derivatives on the behavior of non-stressed and stressed rats were studied using the EZM test. The mean values of the behavioral parameters in the EZM test and the results of the ANOVA are summarized in Supplementary Table S1. A two-way ANOVA revealed a significant STRESS × PEPTIDE interaction for the time spent in the enclosed sector-2 and the percentage of entries in the enclosed sector-2 (F2,52 = 3.25; η2 = 0.12; *p* = 0.047 and F2,52 =3.42; η2 = 0.18; *p* = 0.042, respectively), and a marginally significant STRESS × PEPTIDE interaction for the enclosed sector-1 time (F2,52 = 3.12; η2 = 0.11; *p* = 0.053), without any significant main effects of the factors (F < 1.0; η2 < 0.02; *p* > 0.40). Post hoc analysis revealed that time spent in the open sectors (Figure 1a), time spent in enclosed sector-1 (Figure 1b), time spent in enclosed sector-2 (Figure 1c), and the percentage of entries in enclosed sector-2 (Figure 1d) in non-stressed rats that were administered peptides did not differ from non-stressed control rats (*p* > 0.05). Vehicle-treated rats exposed to ARS demonstrated a significantly increased time spent in enclosed sector-2 (*p* = 0.048; d = 0.96; Figure 1c), and the percentage of entries into enclosed sector-2 (*p* = 0.022; d = 1.47; Figure 1d), and marginally decreased time spent in enclosed sector-1 (*p* = 0.061; d = 0.81; Figure 1b) compared with non-stressed controls. Pretreatment with Semax attenuated the effects of ARS: the percentage of enclosed sector-2 entries in the STR-S group was significantly lower than in the STR-V group (*p* = 0.048; d = 1.48; Figure 1d). Pretreatment with ACTH(6–9)PGP prevented ARS-induced behavioral alterations: time spent in enclosed sector-1 was significantly higher (*p* = 0.044; d = 1.05; Figure 1b), while time spent in enclosed sector-2 (*p* = 0.037; d = 1.12; Figure 1c) and the percentage of entries into enclosed sector-2 (*p* = 0.048; d = 1.45; Figure 1d) were significantly lower in the STR-A group than in the STR-V group.

Thus, the ARS protocol did not induce any significant changes in the conventional indices of anxiety (time spent in, and number of entries into, the open sectors) locomotor activity, and exploration (number of sector entries, rearing, and head-dipping) in rats in the EZM test, 4 h after ARS exposure. However, some behavioral alterations were observed in rats subjected to ARS after vehicle administration: they visited the enclosed sector-2 more often than non-stressed rats, i.e., made more frequent crossings of the open sectors. The peptide pretreatment protected against these alterations in the EZM test.

### 2.2. RNA-Seq Analysis of the Effect of Semax and ACTH(6–9)PGP on the Hippocampal Transcriptome of Non-Stressed Rats

We performed RNA-Seq analysis to determine the effect of Semax and ACTH(6–9)PGP treatment on the hippocampal transcriptome of non-stressed rats. From an analysis of more than 17,000 genes, we did not detect any DEGs in NS-S vs. NS-V at 6 h after Semax administration. We detected only 2 genes (Gabrg1 and Cfh) that had more than a 1.5-fold difference in expression in the NS-A vs. NS-V groups (*p*-values adjusted using the Benjamini–Hochberg procedure (*Padj*) < 0.05) at 6 h after ACTH(6–9)PGP administration. Thus, the *Gabrg1* gene, which encodes the γ1 γ-aminobutyric acid (GABA)-A receptor subunit, was upregulated 2.65-fold, while the *Cfh* gene, which encodes a complement factor, was upregulated 1.68-fold. 

### 2.3. RNA-Seq Analysis of the Effect of ARS on the Hippocampal Transcriptome

**Figure 2:**
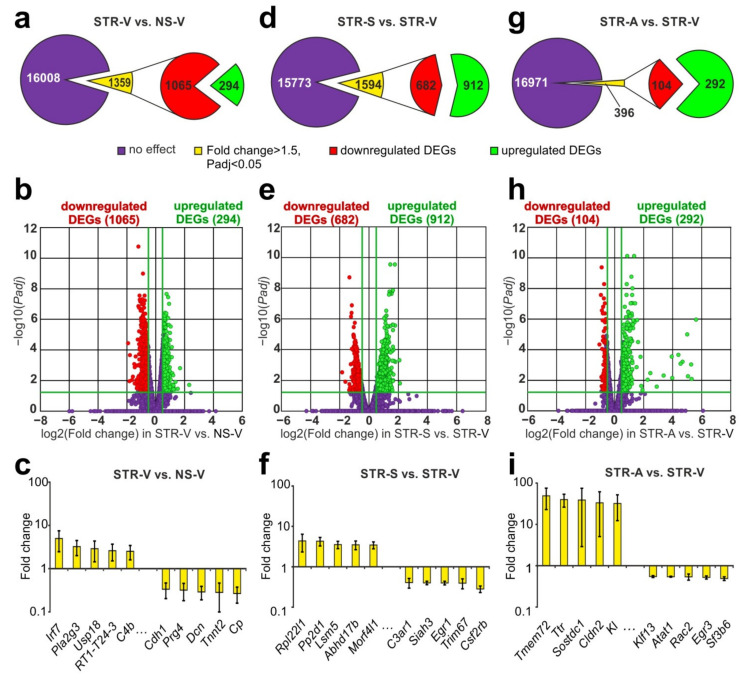
RNA-Seq analysis of the effect of ARS, and Semax or ACTH(6–9)PGP treatment in combination with ARS on the hippocampal transcriptome. (**a**,**d**,**g**) RNA-Seq results presented are for STR-V vs. NS-V (**a**), STR-S vs. STR-V (**d**), and STR-A vs. STR-V (**g**). The numbers in the diagram sectors indicate the number of DEGs. The cutoff for gene expression changes was a 1.50-fold change. Only those genes with *p*-values adjusted using the Benjamini–Hochberg procedure (*Padj*) < 0.05 were selected for analysis. (**b**,**e**,**h**) Volcano plots illustrate the differences in mRNA expression between the STR-V and NS-V groups (**b**), STR-S and STR-V groups (**e**), and the STR-A and STR-V groups (**h**). Up- and downregulated DEGs are represented as green and red dots, respectively (fold change > 1.50; *Padj* < 0.05). Genes that were not differentially expressed are represented as dark purple dots (fold change ≤ 1.50; *Padj* ≥ 0.05). (**c**,**f**,**i**) The top 10 genes that exhibited the greatest fold change in expression in STR-V vs. NS-V (**c**), STR-S vs. STR-V (**f**), and STR-A vs. STR-V (**i**). The data are presented as the mean ± standard error of the mean.

Using RNA-Seq, we assessed the effect of ARS on the mRNA levels of genes in the rat hippocampus at 4.5 h after ARS (STR-V vs. NS-V). We identified 1359 DEGs in STR-V vs. NS-V with 294 upregulated and 1065 downregulated mRNA species (Figure 2a, Supplementary Table S2). A volcano plot illustrates the differences in mRNA expression between the STR-V and NS-V groups (Figure 2b). The top 5, most highly upregulated genes in the rat hippocampus in response to stress were *Irf7*, *Pla2g3*, *Usp18*, *RT1–T24–3*, and *C4b*, while the top 5 downregulated genes were *Cdh1*, *Prg4*, *Dcn, Tnnt2*, and *Cp* (Figure 2c, Supplementary Table S3). We used real-time RT–PCR analysis of the expression of the *Pla2g3*, *Irf7*, *Lrg1*, *Rt1-Ba*, *Drd1*, *Dcn*, and *Tnnt2* genes to verify the RNA-Seq results (Supplementary Figure S1). The real-time RT–PCR results adequately confirmed the RNA-Seq data.

Hierarchical cluster analysis of all DEGs in STR-V vs. NS-V as well as STR-S vs. NS-S, and STR-A vs. NS-A is illustrated in Supplementary Figure S2. The common features of differential expression profile for these comparison groups reflect the effect of ARS. At the same time, individual differences between the groups characterize the contribution of peptides to the variability of the transcriptional profile under ARS conditions.

### 2.4. RNA-Seq Analysis of the Effect of Semax and ACTH(6–9)PGP on the Effect of ARS on the Hippocampal Transcriptome

In the hippocampus of rats collected 6 h after Semax administration and 4.5 h after ARS (STR-S), we identified 1594 DEGs (912 up- and 682 downregulated; Figure 2d, Supplementary Table S4) vs. vehicle-treated rats (STR-V). A volcano plot illustrates the differences in mRNA expression between the STR-S and STR-V groups (Figure 2e). The top 5 most highly upregulated genes in STR-S vs. STR-V were *Rpl22l1*, *Pp2d1*, *Lsm5*, *Abhd17b*, and *Morf4l1*, while the top 5 most markedly downregulated genes were *C3ar1*, *Siah3*, *Egr1*, *Trim67*, and *Csf2rb* (Figure 2f, Supplementary Table S5).

Simultaneously, in the hippocampus of rats subjected to ARS after ACTH(6–9)PGP administration, we identified 396 DEGs (292 upregulated and 104 downregulated) compared with the ARS and vehicle-treated group (Figure 2g, Supplementary Table S6). A volcano plot illustrates the differences in mRNA expression between the STR-A and STR-V groups (Figure 2h). The top 5 upregulated genes were *Tmem72*, *Ttr*, *Sostdc1*, *Cldn2*, and *Kl*) and the top 5 downregulated genes were *Klf13*, *Atat1*, *Rac2*, *Egr3*, and *Sf3b6* (Figure 2i, Supplementary Table S7).

### 2.5. Analysis of RNA-Seq Results in Different Comparison Groups

**Figure 3:**
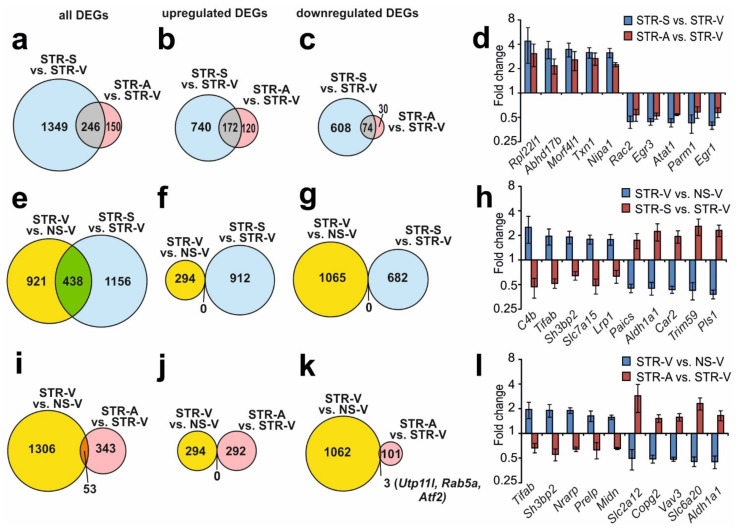
Analysis of RNA-Seq results in different comparison groups. (**a**,**c**,**e**) Schematic comparisons of the results obtained in pairwise comparisons of STR-S vs. STR-V, and STR-A vs. STR-V (**a**–**c**); STR-V vs. NS-V and STR-S vs. STR-V (**e**–**g**); and STR-V vs. NS-V and STR-A vs. STR-V (**i**–**k**) are represented by Venn diagrams. Comparison for all (**a**,**e**,**i**), or upregulated (**b**,**f**,**j**) or downregulated (**c**,**g**,**k**) DEGs. (**d**,**h**,**l**) The top 10 genes that exhibited the greatest fold change in expression in STR-S vs. STR-V (**d**) or STR-V vs. NS-V (**h**,**l**), and lie within the intersection of the gene sets on the Venn diagram (**a**,**f**,**i**), respectively. The data are presented as the mean ± standard error of the mean. The cutoff of gene expression changes was 1.50-fold. Only those genes with *Padj* < 0.05 were selected for analysis.

An analysis of RNA-Seq results obtained from the pairwise comparisons of STR-S vs. STR-V and STR-A vs. STR-V was conducted, which revealed 1349 and 150 DEGs that were unique for Semax and ACTH(6–9)PGP action, respectively. Nevertheless, both peptides altered the mRNA levels of 246 genes under ARS conditions (Figure 3a, Supplementary Table S8). Venn diagrams including only upregulated DEGs and only downregulated DEGs detected under both conditions are illustrated in Figure 3b,c. Analysis identified 172 DEGs that were upregulated under both conditions (Figure 3b) and 74 DEGs that were downregulated under both conditions (Figure 3c). Thus, all DEGs present in the STR-S vs. STR-V and STR-A vs. STR-V comparisons altered their expression unidirectionally under both conditions. The top 10 overlapping genes with the greatest fold change in STR-S vs. STR-V groups are presented in Figure 3d. Individual data points for Figure 3d are provided in Supplementary Table S9. Both Semax and ACTH(6–9)PGP treatment in combination with ARS increased the expression of some genes (*Rpl22l1*, *Abhd17b*, *Morf4l1*, *Txn1*, *Nipa1*, etc.), and decreased the expression levels of other genes (*Rac2*, *Egr3*, *Atat1*, *Parm1*, *Egr1*, etc.) (Supplementary Table S8).

Our analysis revealed that both peptides modulated the expression profile of genes that had been altered by ARS relative to non-stressed rats. There were 438 overlapping DEGs in the STR-S vs. STR-V and STR-V vs. NS-V groups (Figure 3e, Supplementary Table S10). Venn diagrams with only upregulated DEGs and only downregulated DEGs under both conditions are presented in Figure 3f,g. Notably, there were no DEGs that were upregulated under both conditions (Figure 3f), and no DEGs that were downregulated under both conditions (Figure 3g). Semax initiated gene expression that counteracted the effects of ARS. The top 10 overlapping genes with the greatest fold change in STR-S vs. STR-V are presented in Figure 3h. Individual data points for Figure 3h are provided in Supplementary Table S11. Semax treatment of rats that were subjected to ARS upregulated genes that otherwise displayed reduced expression after ARS (*C4b*, *Tifab*, *Sh3bp2*, *Slc7a15*, *Lrp1*, etc.), and vice versa, as Semax downregulated genes that displayed increased expression after ARS (*Paics*, *Aldh1a1*, *Car2*, *Trim59*, *Pls1*, etc.) (Supplementary Table S10).

Our analysis also revealed 50 overlapping DEGs in a comparison of STR-A vs. STR-V and STR-V vs. NS-V (Figure 3i, Supplementary Table S12). Venn diagrams with only upregulated DEGs and only downregulated DEGs under both conditions are presented in Figure 3j,k. Notably, there were no DEGs that were upregulated under both conditions (Figure 3j), and only 3 genes that were simultaneously upregulated in both STR-A vs. STR-V and STR-V vs. NS-V comparisons, (*Utp11l*, which encodes an UTP11-like, U3 small nucleolar ribonucleoprotein; *Rab5a*, which encodes RAB5A, a member of the RAS oncogene family; and *Atf2*, which encodes activating transcription factor 2; Figure 3k). Furthermore, ACTH(6–9)PGP also initiated gene expression that predominantly counteracted the effects of ARS. The top 10 overlapping genes with the greatest fold change in STR-A vs. STR-V are presented in Figure 3l. Individual data points for Figure 3l are provided in Supplementary Table S13. ACTH(6–9)PGP treatment of rats that were subjected to ARS upregulated genes that displayed reduced expression after ARS (*Tifab, Sh3bp2*, *Nrarp*, *Prelp*, *Midn*, etc.), and vice versa, as ACTH(6–9)PGP downregulated genes that displayed increased expression after ARS (*Slc2a12*, *Copg2*, *Vav3*, *Slc6a20*, *Aldh1a1*, etc.) (Supplementary Table S12).

The results of a pairwise comparison of STR-V vs. NS-V, STR-S vs. STR-V, and STR-A vs. STR-V are illustrated in a Venn diagram (Supplementary Figure S3a). We identified 46 genes that were altered by all conditions (Supplementary Table S14). Thus, both Semax and ACTH(6–9)PGP treatment initiated changes in the expression of 46 common genes (*Tifab*, *Sh3bp2*, *Prelp*, *Vdac3*, *Copg2*, etc.) that counteracted the effects of ARS (Supplementary Figure S3b; Table S14) Hierarchical cluster analysis of all DEGs in STR-V vs. NS-V, STR-S vs. STR-V, and STR-A vs. STR-V is illustrated in Supplementary Figure S3c. Semax and ACTH(6–9)PGP induced a correction of gene expression patterns that were disrupted during ARS.

### 2.6. Functional Annotations of DEGs Altered by Acute Stress and MC Peptides

Using the GSEA program, we identified the top 100 signaling pathways associated with DEGs in each of the pairwise comparisons STR-V vs. NS-V, STR-S vs. STR-V, and STR-A vs. STR-V (Supplementary Table S15). Genes that altered their expression after ARS identified in STR-V vs. NS-V were associated with processes of post-translational protein modification and metabolism of RNA (*Eif5a2*, *Eef1a1*, *Rpl5*, *Rpl9*, *Rps3a*, *Cwc27*, *Hnrnpc*, *Sf3a3*, etc.), specific protease processing (*Psma1*, *Psmd6*, *Psma6*, *Psma4*, *Usp47*, *Usp14*, *Usp8*, etc.), vesicle mediated transport (*Exoc6*, *Exoc5*, *Sec22b*, *Trappc13*, *Trappc8*, *Trappc6b*, etc.), immune system function and cytokine signaling (*Psma1*, *Vcam1*, *Map2k4*, *Ppp3ca*, *Calm1*, *Tnfrsf11b*, *Il33*, *Il1r1*, etc.), and the development and function of the nervous system (*Grm5*, *Gabra3*, *Gabra5*, *Gria2*, *Gria3*, *Adam10*, *Skp1*, *Nr3c1*, etc.). Moreover, these DEGs were predominantly downregulated in STR-V vs. NS-V (Supplementary Table S15).

**Figure 4:**
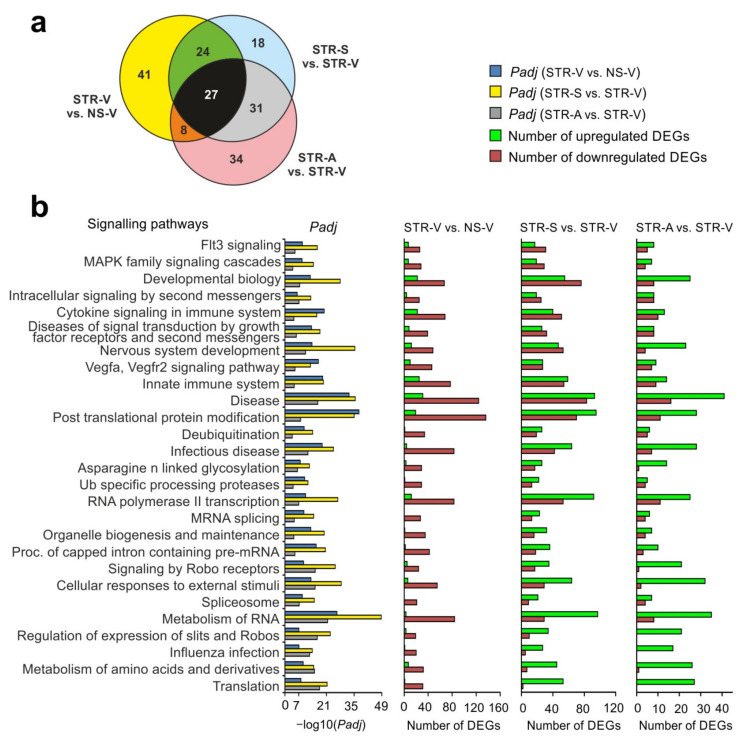
Analysis of the signaling pathways associated with DEGs under ARS and peptide treatment conditions. (**a**) The number of overlapping signaling pathways in the three pairwise comparisons, STR-V vs. NS-V, STR-S vs. STR-V, and STR-A vs. STR-V. (**b**) Functional annotation of DEGs in the three pairwise comparisons, STR-V vs. NS-V, STR-S vs. STR-V, and STR-A vs. STR-V was conducted according to the GSEA database. The number of up- and downregulated DEGs, as well as the *Padj*, are shown. Only those genes and signaling pathways with *Padj* < 0.05 were selected for analysis.

We revealed ~60 overlapping signaling pathways between Semax and ACTH(6–9)PGP action under ARS conditions from the STR-S vs. STR-V and STR-A vs. STR-V pairwise comparisons (Figure 4a). Most of these pathways were associated with upregulated DEGs (Supplementary Table S15). Under ARS conditions, both Semax and ACTH(6–9)PGP altered the expression of genes that were associated with post-translational protein modification, transcription and metabolism of RNA (*Rpl22l1*, *Rps23*, *Rpl9*, *Rps13*, *Rps16*, *Rps14*, *Srsf3*, *Srsf1*, *Tcf7l1*, *Cnot3*, etc.), DNA replication (Pole4, Anapc15, etc.), and the function of the immune (*Cd63*, *Sec61g*, *Psma5*, *Psmd14*, *Psmb4, Hsp90aa1*, etc.) and nervous (*Chrna4*, *Vdac3*, *Grik3*, etc.) systems (Supplementary Table S15).

After additional analysis using the DAVID program, 38 unique functional categories for Semax action were identified that were associated with DEGs in STR-S vs. STR-V, but not in STR-A vs. STR-V (Supplementary Table S16). There were DEGs associated with apoptosis (*Bag6, Pdcd4*, *Pdcl3*, *Tgfbr1*, *Tnfrsf1a*, *Casp3*, *Mapk3*, *Tp53*, *Bcl10*, etc.); regulation of synapses and neurogenesis (*Grm2*, *Gria4*, *Syngap1*, *Gabra2*, *Grin1*, *Synpo*, *Grin2d*, *Chrm4*, *Gabrg1*, *Chrm1*, *Srgap2*, etc.); chromatin regulation (*Hdac2*, *Hdac4*, *Cabin1*, *Smarca4*, etc.); and DNA damage (*Nabp2*, *Cep63*, *Rev1*, etc.). Simultaneously, there were only 2 unique functional categories for ACTH(6–9)PGP action based on the DAVID database, with DEGs encoding ribosomal proteins (*Rps5*, *Rps18*, etc.) and associated with function of the mitochondrion inner membrane (*Etfb*, *Atf2*, *Cox4i1*, *Ddit4*, *G0s2*, etc.).

Based on the GSEA data, there were 27 overlapping signaling pathways from three pairwise comparisons, STR-V vs. NS-V, STR-S vs. STR-V, and STR-A vs. STR-V (Figure 4a). Among them there were signaling pathways related to biogenesis and translation of RNA, and immune and neurotransmitter systems function. We observed that DEGs after ARS that were associated with common signaling pathways were predominantly downregulated in STR-V vs. NS-V (Figure 4b). In contrast, MC peptide treatment predominantly upregulated DEGs that were associated with signaling pathways that overlapped between STR-V vs. NS-V, STR-S vs. STR-V, and STR-A vs. STR-V. Thus, after both Semax and ACTH(6–9)PGP treatment, there were upregulated DEGs that were associated with translation and metabolism of RNA (*Rpl9*, *Eif4a2*, *Rpl7*, *Psma5*), and immune (*Capza2*, *Hsp90aa1*, *Vav3*) and neurotransmission (*Chrna4*, *Vdac3*) systems that were disrupted by ARS (Figure 4b).

## 3. Discussion

Recent studies indicate that multiple peptides are actively involved in the regulation of brain function in response to stress. For example, nociceptin/orphanin FQ (N/OFQ) is an opioid-related neuropeptide that produces increased hypothalamic–pituitary–adrenal (HPA) axis activity, and has been reported to produce both anxiogenic and anxiolytic effects [34,35]. Furthermore, it was shown recently that decreased levels of midbrain and cerebellar nociceptin receptors were associated with less severe symptoms in post-traumatic stress disorder [4]. The role of orexin peptides in stress responses is also established. For example, central injections of orexin in mice evoked sympathetically mediated cardiovascular responses, while conversely, blockade of orexin receptors preferentially reduced the cardiovascular responses to acute psychological stressors [5]. Peptides of the MC family also play an important role in the regulation of stress responses [16,17,36,37]. However, their mechanism of action under stress conditions remains unclear.

The heptapeptide Semax is a synthetic analogue of the ACTH(4–7) fragment, and exerts nootropic, analgesic, and neuroprotective activity [14,15,38,39,40]. In previous studies, Semax administration attenuated cognitive impairment caused by acute stress [16] and diminished the effects of chronic unpredictable stress in rats [17]. In the present study we examined the behavioral effects and molecular genetic mechanisms of Semax and another MC derivative, ACTH(6–9)PGP, in male rats under normal and ARS conditions. Rat behavior was examined using the EZM test, which is widely used for the evaluation of anxiety-related behavior and exploration in rodents [41,42]. The hippocampal transcriptomic effects of the peptides under normal and ARS conditions were examined using RNA-Seq.

In the EZM test, Semax and ACTH(6–9)PGP administration did not affect the behavior of non-stressed rats when examined 5.5 h after treatment. In contrast, in an earlier study, we observed that these peptides exerted anxiolytic-like activity when administrated 15 min before testing [13,43]. Therefore, the anxiolytic effects of the peptides are rapid in onset, but short in duration (less than 5 h). RNA-Seq analysis of the effect of Semax on the hippocampal transcriptome of non-stressed rats revealed no DEGs. Similarly, only 2 genes (*Gabrg1* and *Cfh*), altered their expression in the hippocampus by more than 1.5-fold after ACTH(6–9)PGP treatment under the same conditions. These data are consistent with the absence of behavioral effects of the peptides in non-stressed rats and suggest the relative safety of administration of these peptides.

A growing body of literature shows that acute stress has important effects on the processes of perception and memory acquisition, consolidation, and retrieval [44]. Acute restraint stress has been reported to alter locomotor activity and anxiety-related behaviors in rodents [1,45]. Delayed anxiogenic effects have been described 24–48 h after exposure of rodents to ARS and to a number of different stressful stimuli [46,47,48]. The short-term effects of ARS are not as consistent. An acute 2- or 4-h episode of restraint stress was shown to cause an increase in anxiety-like behavior in rodent 40 min or 2 h after stress exposure [1,49]. But 1-h ARS exposure did not modify mice behavior in elevated plus maze immediately after stress [1,49,50]. Exposure to 2-h restraint stress induced no changes in rat anxiety 1 or 2 h after stress [46]. These conflicting results are probably due to different schedules of stress loading, stress duration, and/or timing of behavioral tests [1,49,50]. In the present study ARS procedure did not cause alterations in the conventional indices of anxiety—the percentage of open sectors time and entries. So, our data indicate a lack of effect of ARS protocol used on the level of anxiety-like behavior 4 h after stress exposure. Probably, the absence of the anxiogenic effect of is due to insufficient stress duration.

Although there were no changes in the anxiety indices in EZM, some stress-induced behavior alterations were recorded in rats 4 after ARS exposure in present experiment. We have observed that control unstressed rats spent more time in the “home sector” with a low number of transitions between both protected sectors. However, a higher number of transitions between both protected sectors were observed in the EZM test in vehicle-treated stressed rats, as compared to non-stressed control. The total number of sector entries, which is used as a measure of locomotor activity on the maze, was not affected by stress. Earlier, we have studied the anxiety level in rats 30 min after exposure to the same ARS protocol. It was shown that stressed rats had an increased percentage of open arm entries, and time spent in the open arms in the elevated plus maze (EPM), compared to control [16]. Taken together, our results indicate that the ARS procedure used led to altered emotional behavior 30 min and 4 h after stress exposure. The alterations in the aforementioned behavioral indices may also indicate hyperactivity, high impulsivity and/or high escape motivation [51]. The behavior of stressed rats is characterized by a high level of behavioral reactivity, directed towards rapid escape from the apparatus [52]. Such behaviors, also considered manifestations of impulsivity and disinhibition, are well-known consequences of different chronic stress procedures in rodents [50,53]. Discrepancies between our results and others may be due to the different stress paradigms employed. For example, in present experiment the rats were restraint under a more stressful environment (bright light and acoustic load). A strong stressful stimulus, such as bright light, is sufficient to induce hyperactivity and impulsive reactions [51].

Using RNA-Seq, we identified 1359 DEGs (>1.5-fold change) at 4.5 h after ARS. An analysis of the expression of 7 genes by real-time RT–PCR confirmed the RNA-Seq results. Our analysis revealed a major downregulation of gene expression under ARS conditions, and a functional annotation of the DEGs was conducted. ARS significantly reduced the expression of a large number of genes associated with the regulation of biogenesis and metabolism of RNA and protein. A decrease in the expression of genes encoding the structural proteins of ribosomes (*Rpl5*, *Rpl9*, *Rps3a*, etc.) was observed, and the assembly of ribosomes and the translation of proteins are well known to be consistently but subtly regulated. Any failure in this process could profoundly slow the growth and development of cells [54]. Additionally, DEGs related to neurotransmitter systems (*Grm5*, *Gabra3*, *Gabra5*, *Gria2*, *Gria3*, *Adam10*, *Skp1*, *Nr3c1*, etc.) were downregulated under ARS conditions. These data are consistent with a decrease in neuronal activity under conditions of depression [55,56]. Previously, using high-throughput RNA sequencing, a significant downregulation of genes encoding neurotransmitter system components (GABAergic and dopaminergic synapses, proteins used in the formation and functioning of synaptic vesicles, axonal growth, and amphetamine- and morphine-dependent processes) was observed under conditions of stress-induced depression in the medial prefrontal cortex in mice [57].

We also identified a significant change in the expression of genes of the immune system and cytokine signaling (*Psma1*, *Vcam1*, *Map2k4*, *Ppp3ca*, *Calm1*, *Tnfrsf11b*, *Il33*, *Il1r1*, etc.) under ARS conditions, in line with a large number of studies that suggest a role for the immune system in the stress response [58,59,60,61]. Clinical studies reveal that depression is associated with elevated levels of IL-6 and IL-1β proteins in the cerebrospinal fluid [62,63]. Recently, acute stress was also shown to cause an increase in the number of T cells that produce IL4, IL-5, and IL-10 [64]. Finally, Dygalo et al. (2017) reported that acute stress increased the mRNA level of the anti-apoptotic molecule of B cells, Bcl-xL, in the hippocampus [65].

In the present study, rats that received injections of Semax or ACTH(6–9)PGP 30 min before ARS exposure demonstrated behavior in the EZM test similar to that of non-stressed vehicle-treated rats. These findings suggest the peptides attenuated ARS-induced behavioral alterations, without having an effect in non-stressed rats, consistent with the proposed anti-stress behavioral effects of the MC derivatives.

Using RNA-Seq, we identified more than 1500 DEGs 6 h after Semax administration in the rat hippocampus under ARS conditions. Simultaneously, there were about 400 DEGs observed after ACTH(6–9)PGP treatment under the same conditions. In contradistinction to ACTH(6–9)PGP, Semax actively modulated gene expression associated with apoptosis, regulation of synapses, neurogenesis, and chromatin, and DNA damage. Similarly, for ACTH(6–9)PGP action, unique DEGs were associated with ribosomal proteins and the function of the mitochondrial inner membrane. These different effects of Semax and ACTH(6–9)PGP peptides on the transcriptome may be due to the different structural organization of these MC analogues. Nevertheless, 246 overlapping DEGs were identified, and expression of these DEGs was changed unidirectionally by both peptides under ARS conditions. Moreover, we found that about 60% of signaling pathways identified were common to both peptides under ARS conditions. Among them were signaling pathways for biogenesis and translation of RNA, DNA replication, and functioning of the immune and nervous systems. Additionally, functional analysis of DEGs revealed that the action of the synthetic MC derivatives was associated with modulation of the activity of a number of metabolic systems that were affected by stress. In particular, genes for biogenesis and translation of RNA were upregulated by the peptides. Notably, the activation of the expression of genes related to the synthesis of ribosomes by Semax was previously observed under permanent middle cerebral artery occlusion (pMCAO) conditions [66]. However, here this effect was observed against a background of a decrease in the expression of ribosomal protein genes in the hippocampus from rats subjected to ARS conditions compared with non-stressed rats. Simultaneously, both Semax and ACTH(6–9)PGP predominantly upregulated DEGs related to the functioning of the immune and nervous systems that were downregulated under ARS conditions in STR-V vs. NS-V. Thus, both Semax and ACTH(6–9)PGP initiated gene expression that counteracted the effects of ARS. Here, we identified 438 and 53 such genes, respectively, and 46 of these DEGs (*Rpl7*, *Rpl9*, *Eif4a2*, *Psma5*, *Hsp90aa1*, *Tifab*, *Sh3bp2*, *Prelp*, *Vdac3*, *Copg2*, etc.) are common for both peptide treatments. In a previous study, a compensatory effect of Semax was observed on the expression of genes altered by experimental cerebral ischemia [67]. Semax compensated for changes in the expression of the genes encoding neurotrophins and their receptors in the frontal cortex and hippocampus after pMCAO [67]. Moreover, in the first few hours after the onset of global cerebral ischemia in rats, Semax had an effect opposite to that of ischemia on the expression of *Vegfa* mRNA [68]. Semax also modulates the expression of genes related to the activity of immune cells after pMCAO [66,69]. Following the transient middle cerebral artery occlusion (tMCAO) model, Semax suppressed the expression of genes related to inflammatory processes and activated the expression of genes related to neurotransmission [70]. In contrast, ischemia-reperfusion alone activated the expression of inflammation-related genes and suppressed the expression of neurotransmission-related genes [71]. In the present study, the normalizing effect of MC derivatives on ARS-induced behavioral alterations may be associated with a correction of gene expression patterns that are disrupted during ARS conditions.

**Figure 5:**
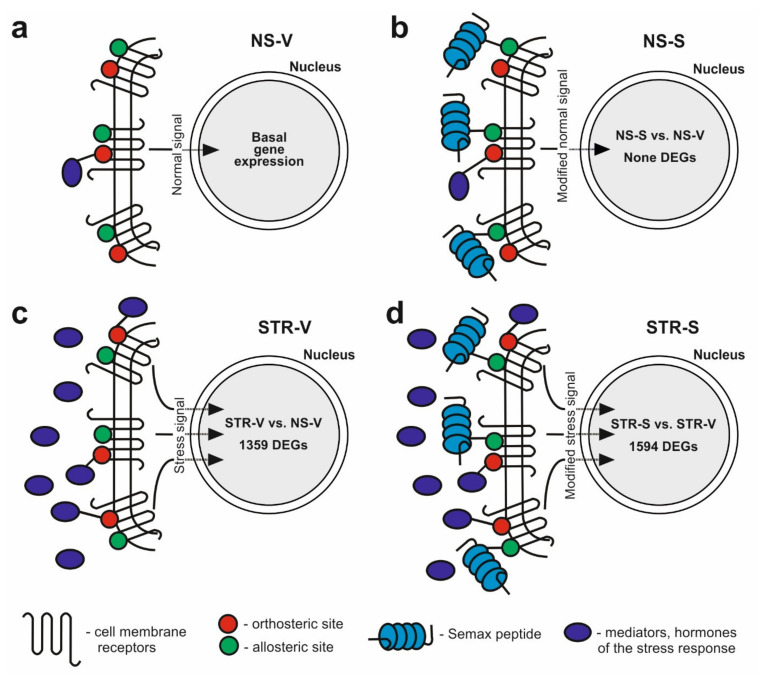
A model for the formation of a transcriptome response under normal and ARS conditions, and after MC peptide (e.g., Semax) administration. Four situations are considered: (**a**) non-stressed conditions without peptide treatment (NS-V); (**b**) Semax peptide action under non-stressed conditions (NS-S); (**c**) ARS conditions without peptide treatment (STR-V); (**d**) Semax peptide action under ARS conditions (STR-S).

Recent studies demonstrate that the mechanism of action of peptides is not only associated with their orthosteric binding with receptors [72,73]. Much attention is being paid to the allosteric mechanisms in these interactions. Such allosteric binding leads to many different specific cell responses and explains the multi-functionality of peptides [28]. Our results revealed the general and specific effects of MC peptides in the regulation of gene expression under normal and ARS conditions. Moreover, based on our RNA-Seq data, we proposed a model for the formation of a transcriptome response after MC peptide administration based on the allosteric binding of peptides with receptors (Figure 5). Normally, the interaction of regulatory molecules with their receptors ensures signal transmission to the nucleus and the expression of the corresponding target genes (Figure 5a). MC peptide administration was accompanied by little or no changes in gene expression in the hippocampus under non-stressed conditions (Figure 5b). This may be due to the weak allosteric binding of the peptides with receptors and the corresponding response of these receptors to the received signal. Under ARS conditions, the products of the sympathoadrenal and HPA systems are released in large quantities [74]. As a result, many regulatory compounds, including hormones and neurotransmitters, interact with their own receptors in an orthosteric manner. This leads to the transmission of a powerful signal to the cell nucleus to modulate the expression of a large number of genes. Indeed, in our experiments, we detected 1359 DEGs in response to ARS (Figure 5c). In the presence of Semax and ARS, we detected an even larger number of DEGs (1594 DEGs) than in response to ARS without the peptide (Figure 5d). It is possible that such a result is related to allosteric interactions of the peptide with various receptors. Moreover, some of these receptors might be involved in orthosteric binding with regulatory compounds released as part of the stress response. As a result, the expression of a defined set of genes can be linked to the presence of a peptide due to changes in the affinity of ligand binding to the receptor. Simultaneously, the expression of individual genes that are not involved in stress can be modulated after peptide administration (Figure 3e). Indeed, under ARS conditions, Semax altered the expression of about 450 genes that were regulated by ARS and not in non-stressed rats. Simultaneously, the expression of more than 900 genes that were not regulated by stress were modulated by Semax administration.

A limitation of our study is the analysis of the expression of only mRNAs; the genome-wide noncoding RNAs expression analysis may reveal some additional regulatory axes involved in the response after MC peptide administration under normal and ARS conditions. The combined study of mRNAs and noncoding RNAs (microRNAs, circular RNAs, etc.) changes under the influence of the MC peptides and would further elucidate the mechanisms associated with stress in various brain structures.

## 4. Materials and Methods

### 4.1. Animals

Male Wistar rats weighing 220–250 g were obtained from the Stolbovaya Breeding Center (Moscow region, Russia). A total of 58 rats were used in this study. Rats were housed in plastic cages (*n* = 5–6) at room temperature and were maintained on a 12 h light/dark cycle (lights on at 08:00). Food and water were available ad libitum. All rats were adapted (including daily handling) to their housing environment for at least 1 week before experimentation. For the behavioral tests, the rats were randomly divided into six experimental groups, each group consisting of 9–10 rats, as described below: (1) Non-stressed + Vehicle (NS-V); (2) Non-stressed + Semax (NS-S); (3) Non-stressed + ACTH(6–9)PGP (NS-A); (4) Stress + Vehicle (STR-V); (5) Stress + Semax (STR-S); (6) Stress + ACTH(6–9)PGP (STR-A).

### 4.2. Experimental Design

The experiments were performed during the light period of the light/dark cycle (between 10 a.m. and 6 p.m.). The peptides were administrated 30 min prior to stress procedure, because we have shown earlier that Semax injected 30 min before stress attenuated cognitive impairment caused by ARS [16]. 30 min after the peptides administration, rats were kept in their home cages or submitted to ARS for 1 h. Four hours after ARS termination, the animals were subjected to the behavioral test (elevated zero maze) and then were euthanized 30 min later to collect hippocampal samples. The time point post ARS (4–4.5 h) was chosen to study prolong behavioral and transcriptional ARS effects. It has been shown that ARS induced alterations in cognition, behavior, and hippocampal gene expression take place between 1 and 24 h after acute stress [47,75].

### 4.3. Peptide Administration

The peptides, Semax and ACTH(6–9)PGP, were synthesized at the Institute of Molecular Genetics, Russian Academy of Sciences (Moscow, Russia). The peptides were dissolved in sterile distilled water. NS-V and STR-V groups received intraperitoneal (i.p.) injections of vehicle (sterile water, 1 mL/kg body weight). NS-S and STR-S groups received i.p. injections of Semax peptide (100 μg/kg), and NS-A and STR-A groups received i.p. injections of ACTH(6–9)PGP peptide (100 μg/kg). The Semax dosage and administration route were chosen based on the our previous studies demonstrating a positive Semax effects after i.p and intranasal administration in the 50–150 μg/kg dose range [14,15]. The neuroprotective action of Semax at dose 100 μg/kg was demonstrated in the model of ischemia-reperfusion in rats [70]. The ACTH(6–9)PGP was used in dosage the equal as Semax.

### 4.4. Acute Restraint Stress

Restraint stress merges the emotional and physical aspects of stress, and the model is widely used to study the effects of acute and chronic stress exposure in rats [45]. In our study, 30 min after drug administration, rats in all stress groups (STR-V, STR-S, STR-A) were subjected to ARS. Each rat was submitted to restraint by placing it into a cylindrical plastic restrainer (165 × 55 × 55 mm; OpenScience, Moscow region, Russia), ventilated with holes. The restrainer allowed only limited lateral movements without causing pain. ARS was caused by the restraint combined with bright lighting (500 lx) and intermittent acoustic exposure (sounding of an electric bell, 80 dB) for 60 min. After the ARS procedure, rats were returned to their home cages. Non-stressed rats were left undisturbed in their home cages throughout the stress procedure.

### 4.5. Behavioral Assessment

All rats were subjected to an elevated zero-maze test (EZM) 5.5 h after vehicle or peptide injection (4 h after ARS). The EZM (OpenScience, Moscow region, Russia) consisted of a gray plastic annular runway (width 10 cm, outer diameter 105 cm) elevated 70 cm above the floor. The two opposing 90° sectors were protected by 27 cm high inner and outer walls, while the remaining two sectors had no walls. The maze was illuminated by diffuse indirect room light (50 lx). Each rat was placed in the protected sector (protected sector-1) and observed for 5 min. After each test, the EZM was cleaned with 70% ethanol. The following parameters were analyzed: number of entries into and total time spent in the open sectors, number of entries into and total time spent in each of the enclosed sectors, the number of rears and head dips. The percentage of enclosed sector-2 entries (100 × enclosed sector-2/total enclosed sectors entries) was calculated for each rat.

### 4.6. Statistical Analysis of the Behavioral Data

Behavioral data were analyzed using STATISTICA 10.0 software (TIBCO Software Inc. CA, USA). Data from behavioral assessment were expressed as mean ± standard error of the mean (M ± SEM) and are presented as a violin plots, showing the distribution of values. Prior to analysis, the normality of the data distribution within each parameter was verified by Kolmogorov-Smirnov test (*p* > 0.05), and homogeneity of variances was assessed with Levene’s test (*p* > 0.05). The results were analyzed using two-way Analysis of Variance (ANOVA) with two between-subject factors. The factors were STRESS (two levels: non-stressed and stressed) and PEPTIDE (three levels: vehicle, Semax and ACTH(6–9)PGP). When appropriate, the post-hoc comparisons were performed with the Dunnett’s multiple comparison test. The effect size was determined by partial ete squared for ANOVA (η2) and Cohen’s d (d) for pair-wise comparison. Statistical differences were considered significant when *p* ≤ 0.05. Violin plots were constructed by BoxPlotR: a web-tool for generation of box plots [76].

### 4.7. Sample Collection and RNA Isolation

Rats were sacrificed 6 h after vehicle/peptide injection (4.5 h after ARS) and hippocampal samples were collected. Rats were decapitated and brains were quickly removed on an ice-cold plate. The hippocampi from left and right hemispheres were isolated and rapidly weighed.

Hippocampal tissues were placed in RNAlater (Ambion, Austin, TX, USA) solution for 24 h at 0 °C and then stored at –70 °C. Total RNA from the hippocampus was isolated using TRI Reagent (MRC, Cincinnati, OH, USA) and acid guanidinium thiocyanate–phenol–chloroform extraction [77]. The isolated RNA was treated with deoxyribonuclease I (DNase I) (Thermo Fisher Scientific Baltics UAB, Vilnius, Lithuania) in the presence of RiboLock ribonuclease (RNase) inhibitor (Thermo Fisher Scientific Baltics UAB, Vilnius, Lithuania), according to the manufacturer’s recommended protocol. Deproteinization was performed using a 1:1 phenol:chloroform mixture. The isolated RNA was precipitated with sodium acetate (3.0 M, pH 5.2) and ethanol. RNA integrity was checked using capillary electrophoresis (Experion, BioRad, Hercules, CA, USA). RNA integrity number (RIN) was at least 9.0.

### 4.8. RNA-Seq

Total RNA isolated from the hippocampus was used in this experiment. The RNA-Seq experiment was conducted with the participation of ZAO Genoanalytika, Moscow, Russia. For RNA-Seq, the polyA fraction of the total RNA was obtained using the oligoT magnetic beads of the Dynabeads^®^ mRNA Purification Kit (Ambion, Austin, TX, USA). cDNA (DNA complementary to RNA) libraries were prepared using the NEBNext^®^ mRNA Library Prep Reagent Set (New England Biolabs, Ipswich, MA USA). The concentration of cDNA libraries was measured using Qbit 2.0 and the Qubit dsDNA HS Assay Kit (Thermo Fisher Scientific, Waltham, MA, USA). The length distribution of library fragments was determined using the Agilent High Sensitivity DNA Kit (Agilent, Santa Clara, CA, USA). Sequencing was carried out using an Illumina HiSeq 1500 instrument. At least 10 million reads (1/50 nt) were generated.

### 4.9. RNA-Seq Data Analysis

Five pairwise comparisons of RNA-Seq results (STR-V vs. NS-V, STR-S vs. STR-V, STR-A vs. STR-V, NS-S vs. NS-V and NS-A vs. NS-V) were used to analyze the action of the peptides Semax and ACTH(6–9)PGP on the transcriptome. Each of the comparison groups (NS-V, NS-S, NS-A, STR-V, STR-S, STR-A) included three animals (*n* = 3). All genes were annotated on the NCBI Reference Sequence database. The levels of gene expression were measured using the DESeq2 program. Only genes that exhibited changes in expression >1.5-fold and had a *p*-values adjusted using the Benjamini–Hochberg procedure lower 0.05 (*Padj* < 0.05) were considered.

### 4.10. cDNA Synthesis 

cDNA synthesis was conducted in 20 μL of reaction mixture containing 2 mg of RNA using the reagents of a RevertAid First Strand cDNA Synthesis Kit (Thermo Fisher Scientific Baltics UAB, Vilnius, Lithuania) in accordance with the manufacturer’s instructions. Oligo(dT)_18_ primers were used to analyse mRNA.

### 4.11. Real-Time Reverse Transcription Polymerase Chain Reaction (RT–PCR)

The 25 μL polymerase chain reaction (PCR) mixture contained 2 μL of 0.2× reverse transcriptase reaction sample, forward and reverse primers (5 pmol each), 5 μL of 5× reaction mixture (Evrogen Joint Stock Company, Moscow, Russia) including PCR buffer, Taq DNA polymerase, deoxyribonucleoside triphosphates (dNTP), and the intercalating dye SYBR Green I. Primers specific to the genes studied were selected using OLIGO Primer Analysis Software version 6.31 (Molecular Biology Insights Inc., Colorado Springs, CO, USA) and were synthesized by the Evrogen Joint Stock Company (Table 1). The amplification of cDNAs was performed using a StepOnePlus Real-Time PCR System (Applied Biosystems, Foster City, CA, USA) in the following mode: stage 1 (denaturation), 95 °C, 10 min; stage 2 (amplification with fluorescence measured), 95 °C, 15 s; 65 °C, 25 s; 72 °C, 35 s (40 cycles).

### 4.12. Data Analysis of Real-Time RT–PCR and Statistics

Reference gene *Gapdh* was used to normalize the cDNA samples [78]. Calculations were performed using BestKeeper, version 1 [79] and Relative Expression Software Tool (REST) 2005 software (gene-quantification, Freising-Weihenstephan, Bavaria, Germany) [80]. The manual at the site “REST.-gene-quantification.info” was used to evaluate expression target genes relative to the expression levels of the reference genes. The values were calculated as Ef^Ct(ref)^/Ef^Ct(tar)^, where Ef is the PCR efficiency, Ct(tar) is the average threshold cycle (Ct) of the target gene, Ct(ref) is the average Ct of the reference gene, and Ef^Ct(ref)^ is the geometric average Ef^Ct^ of the reference genes. PCR efficiencies were assessed using the amplification of a series of standard dilutions of cDNAs and computed using REST software [80]. The efficiency values for all PCR reactions were in the range 1.88 to 2.03 (Table 1). At least four animals were included in each comparison group. When comparing data groups, statistically significant differences were considered with the probability *p* < 0.05. Additional calculations were performed using Microsoft Excel (Microsoft Office 2010, Microsoft, Redmond, Washington, USA).

### 4.13. Functional Analysis

Database for Annotation, Visualization and Integrated Discovery (DAVID v6.8) [81] and Gene Set Enrichment Analysis (GSEA) [82] was used to annotate the functions of the differentially expressed genes. When comparing data groups, statistically significant differences were considered with the probability *p* < 0.05. To control the false discovery rate we used Benjamini–Hochberg procedure. Hierarchical cluster analysis of DEGs was performed using Heatmapper [83]. Volcano plots were constructed by Microsoft Excel (Microsoft Office 2010, Microsoft, Redmond, WA, USA).

### 4.14. Availability of Data and Material

RNA-sequencing data have been deposited in the Sequence Read Archive database under accession code PRJNA633551PRJNA491404 (SAMN14946457-SAMN14946474, [84].

## 5. Conclusions

Thus, a comparative analysis of the changes in the transcriptome profile in the rat hippocampus in response to the administration of Semax and ACTH(6–9)PGP peptides under normal and ARS conditions made it possible to identify individual genes and metabolic systems as targets of the MC peptides. Moreover, a compensatory effect on gene expression of the MC derivatives under acute stress was revealed. Based on the transcriptome analysis, a model of peptide regulation of the brain under normal and stress conditions was proposed.

## Figures and Tables

**Table 1:** The characterization of the primers for real-time RT-PCR.

Gene Symbol	Primer (5’-3’)	RefSeq ID	Amplicon Size (bp)	Effectiveness ± Standard Error
*Tnnt2*	F: AACAGGAGGAAGGCTGAAGAT	NM_012676	278	1.88 ± 0.03
	R: TATTTCTGCTGCTTGAACTT			
*Pla2g3*	F: CAAGTTCCACCTGCTCAACA	NM_001106015	207	2.02 ± 0
	R: GTGCCTTTATCCCAGAAATG			
*Lrg1*	F: ACTTGGACCTGGCGGAGAA	NM_001009717.1	108	1.9 ± 0.05
	R: GCAGCGACTCAAGCAGGTT			
*Rt1-Ba*	F: TGTGGAGGTCAAGACGACATT	NM_001008831	344	1.92 ± 0.03
	R: AAAGCAGATGAGGGTGTT			
*Drd1*	F: CATAGAGACGGTGAGCATTA	NM_012546	251	1.96 ± 0
	R: TGTGTGTGACAGGTTGGAT			
*Irf7*	F: ATACTCCCATCTTTGACTTC	NM_001033691	230	1.98 ± 0.04
	R: ACTGCTGCTGTCCAGAGA			
*Dcn*	F: AATGGCAGTCTGGCTAATGT	NM_024129	250	2.02 ± 0.03
	R: TGAAGGTGTGTGGGTGAAT			
*Gapdh*	F: ACTCTACCCACGGCAAGTTCAACG	NM_017008.4	148	2.01 ± 0.03
	R: GTAGACTCCACGACATACTCAGCAC			

## Data Availability

Publicly available datasets were analyzed in this study. These data can be found here: [84].

## Supplementary Materials

The following are available online at https://www.mdpi.com/article/10.3390/ijms221810054/s1.

## References

[1] Domingues M., Casaril A.M., Birmann P.T., Bampi S.R., Lourenço D.D.A., Vieira B.M., Dapper L.H., Lenardao E.J., Sonego M., Collares T. (2019). Effects of a selanylimidazopyridine on the acute restraint stress-induced depressive- and anxiety-like behaviors and biological changes in mice. Behav. Brain Res..

[2] Schwabe L. (2016). Memory under stress: From single systems to network changes. Eur. J. Neurosci..

[3] Floriou-Servou A., von Ziegler L., Stalder L., Sturman O., Privitera M., Rassi A., Cremonesi A., Thöny B., Bohacek J. (2018). Distinct Proteomic, Transcriptomic, and Epigenetic Stress Responses in Dorsal and Ventral Hippocampus. Biol. Psychiatry.

[4] Narendran R., Tollefson S., Fasenmyer K., Paris J., Himes M.L., Lopresti B., Ciccocioppo R., Mason N.S. (2019). Decreased Nociceptin Receptors Are Related to Resilience and Recovery in College Women Who Have Experienced Sexual Violence: Therapeutic Implications for Posttraumatic Stress Disorder. Biol. Psychiatry.

[5] Carrive P. (2017). Orexin, Stress and Central Cardiovascular Control. A Link with Hypertension?. Neurosci. Biobehav. Rev..

[6] Kolomin T.A., Agapova T.I., Agniullin I.V., Shram S.I., Shradina M.I., Slominskiĭ P.A., Limborskaia S.A., Miasoedov I.F. (2013). Transcriptome alteration in hippocampus under the treatment of tuftsin analog Selank. Zhurnal Vyss. Nervn. Deiatelnosti Im. I P Pavlov..

[7] Koenig S., Luger T.A., Scholzen T.E. (2006). Monitoring neuropeptide-specific proteases: Processing of the proopiomelanocortin peptides adrenocorticotropin and α-melanocyte-stimulating hormone in the skin. Exp. Dermatol..

[8] Wikberg J.E. (1999). Melanocortin receptors: Perspectives for novel drugs. Eur. J. Pharmacol..

[9] Giuliani D., Ottani A., Neri L., Zaffe D., Grieco P., Jochem J., Cavallini G.M., Catania A., Guarini S. (2017). Multiple beneficial effects of melanocortin MC4 receptor agonists in experimental neurodegenerative disorders: Therapeutic perspectives. Prog. Neurobiol..

[10] Zhou Y., Cai M. (2017). Novel approaches to the design of bioavailable melanotropins. Expert Opin. Drug Discov..

[11] Eipper B.A., Mains R.E. (1980). Structure and Biosynthesis of Pro-Adrenocorticotropin/Endorphin and Related Peptides. Endocr. Rev..

[12] Fridmanis D., Roga A., Klovins J. (2017). ACTH receptor (MC2R) specificity: What do we know about underlying molecular mechanisms?. Front. Endocrinol..

[13] Levitskaya N.G., Glazova N.Y., Sebentsova E.A., Manchenko D.M., Andreeva L.A., Kamensky A.A., Myasoedov N.F. (2019). Nootropic and anxiolytic effects of heptapeptide ACTH6-9Pro-Gly-Pro. Russ. J. Physiol..

[14] Levitskaya N.G., Glazova N.Y., Sebentsova E.A., Manchenko D.M., Vilensky D.A., Andreeva L.A., Kamensky A.A., Myasoedov N.F. (2008). Investigation of the Spectrum of Physiological Activities of the Heptapeptide Semax, an ACTH 4–10 Analogue. Neurochem. J..

[15] Ashmarin I., Nezavibatko V., Levitskaya N., Koshelev V., Kamensky A. (1995). Design and Investigation of an ACTH(4-10) Analog Lacking D-Amino Acids and Hydrophobic Radicals. Neurosci. Res. Commun..

[16] Glazova N.Y., Sebentsova E.A., Manchenko D.M., Andreeva L.A., Dergunova L.V., Levitskaya N.G., Limborska S.A., Myasoedov N.F. (2018). The Protective Effect of Semax in a Model of Stress-Induced Impairment of Memory and Behavior in White Rats. Biol. Bull..

[17] Yatsenko K.A., Glazova N.Y., Inozemtseva L.S., Andreeva L.A., Kamensky A.A., Grivennikov I.A., Levitskaya N., Dolotov O.V., Myasoedov N.F. (2013). Heptapeptide semax attenuates the effects of chronic unpredictable stress in rats. Dokl. Biol. Sci..

[18] Potaman V., Alfeeva L., Kamensky A., Nezavibatko V. (1993). Degradation of ACTH/MSH(4–10) and its synthetic analog semax by rat serum enzymes: An inhibitor study. Peptides.

[19] Shevchenko K.V., Dulov S.A., Andreeva L.A., Nagaev I.Y., Shevchenko V.P., Radilov A.S., Myasoedov N.F. (2016). Stability of His-Phe-Arg-Trp-Pro-Gly-Pro to Leucine Aminopeptidase, Carboxypeptidase Y, and Rat Nasal Mucus, Blood, and Plasma. Russ. J. Bioorg. Chem..

[20] Potaman V., Antonova L., Dubynin V., Zaitzev D., Kamensky A., Myasoedov N., Nezavibatko V. (1991). Entry of the synthetic ACTH(4–10) analogue into the rat brain following intravenous injection. Neurosci. Lett..

[21] Vyunova T.V., Shevchenko K.V., Shevchenko V.P., Bobrov M.Y., Bezuglov V.V., Myasoedov N.F. (2006). Binding of Regulatory Neuropeptide [3H] Semax, Labeled in Terminal Pro, to Plasma Membranes of the Rat Forebrain. Neurochem. J..

[22] Shevchenko K.V., Nagaev I.Y., Babakov V.N., Andreeva L.A., Shevchenko V.P., Radilov A.S., Myasoedov N.F. (2015). Proteolysis of His-Phe-Arg-Trp-Pro-Gly-Pro in the blood and brain of rats in vivo. Dokl. Biochem. Biophys..

[23] Dores R.M., Liang L., Davis P., Thomas A.L., Petko B. (2016). 60 YEARS OF POMC: Melanocortin receptors: Evolution of ligand selectivity for melanocortin peptides. J. Mol. Endocrinol..

[24] Hruby V.J., Wilkes B.C., Hadley M.E., Al-Obeidi F., Sawyer T.K., Staples D.J., Devaux A.E., Dym O., Castrucci A.M. (1987). alpha-Melanotropin: The minimal active sequence in the frog skin bioassay. J. Med. Chem..

[25] Clark A.J., Forfar R., Hussain M., Jerman J., McIver E., Taylor D., Chan L. (2016). ACTH antagonists. Front. Endocrinol..

[26] Todorovic A., Lensing C.J., Holder J.R., Scott J.W., Sorensen N.B., Haskell-Luevano C. (2018). Discovery of Melanocortin Ligands via a Double Simultaneous Substitution Strategy Based on the Ac-His-dPhe-Arg-Trp-NH2 Template. ACS Chem. Neurosci..

[27] Palmer D., Gonçalves J.P.L., Hansen L.V., Wu B., Hald H., Schoffelen S., Diness F., Le Quement S.T., Nielsen T.E., Meldal M. (2017). Click-Chemistry-Mediated Synthesis of Selective Melanocortin Receptor 4 Agonists. J. Med. Chem..

[28] Vyunova T.V., Andreeva L.A., Shevchenko K.V., Myasoedov N.F. (2016). Synacton and individual activity of synthetic and natural corticotropins. J. Mol. Recognit..

[29] Dergunova L.V., Filippenkov I.B., Limborska S.A., Myasoedov N.F. (2020). Pharmacotranscriptomics of peptide drugs with neuroprotective properties. Med. Res. Rev..

[30] An T., He Z.-C., Zhang X.-Q., Li J., Chen A.-L., Tan F., Chen H.-D., Lv B.-H., Lian J., Gao S.-H. (2019). Baduanjin exerts anti-diabetic and anti-depression effects by regulating the expression of mRNA, lncRNA, and circRNA. Chin. Med..

[31] Kozlovskii I.I., Danchev N.D. (2003). The optimizing action of the synthetic peptide Selank on a conditioned active avoidance reflex in rats. Neurosci. Behav. Physiol..

[32] Kasian A., Kolomin T., Andreeva L., Bondarenko E., Myasoedov N., Slominsky P., Shadrina M. (2017). Peptide Selank Enhances the Effect of Diazepam in Reducing Anxiety in Unpredictable Chronic Mild Stress Conditions in Rats. Behav. Neurol..

[33] Volkova A., Shadrina M., Kolomin T., Andreeva L., Limborska S., Myasoedov N., Slominsky P. (2016). Selank Administration Affects the Expression of Some Genes Involved in GABAergic Neurotransmission. Front. Pharmacol..

[34] Green M.K., Barbieri E.V., Brown B.D., Chen K.-W., Devine D.P. (2007). Roles of the bed nucleus of stria terminalis and of the amygdala in N/OFQ-mediated anxiety and HPA axis activation. Neuropeptides.

[35] Zhang Y., Gandhi P.R., Standifer K.M. (2012). Increased Nociceptive Sensitivity and Nociceptin/Orphanin FQ Levels in a Rat Model of PTSD. Mol. Pain.

[36] Yamano Y., Yoshioka M., Toda Y., Oshida Y., Chaki S., Hamamoto K., Morishima I. (2004). Regulation of CRF, POMC and MC4R Gene Expression after Electrical Foot Shock Stress in the Rat Amygdala and Hypothalamus. J. Vet. Med. Sci..

[37] Markov D.D., Yatsenko K.A., Inozemtseva L.S., Grivennikov I.A., Myasoedov N.F., Dolotov O.V. (2017). Systemic N-terminal fragments of adrenocorticotropin reduce inflammation- and stress-induced anhedonia in rats. Psychoneuroendocrinology.

[38] Asmarin I.P., Nezavibat’ko V.N., Miasoedov N.F., Kamenskiĭ A.A., Grivennikov I.A., Ponomareva-Stepnaia M.A., Andreeva L.A., Kaplan A.I., Koshelev V.B., Riasina T. (1997). V [A nootropic adrenocorticotropin analog 4-10-semax (l5 years experience in its design and study)]. Zhurnal Vyss. Nervn. Deiatelnosti Im. I P Pavlov..

[39] Kaplan A.Y., Kochetova A.G., Nezavibathko V.N., Rjasina T.V., Ashmarin I.P. (1996). Synthetic ACTH analogue semax displays nootropic-like activity in humans. Neurosci. Res. Commun..

[40] Manchenko D.M., Glazova N.Y., Levitskaya N., Andreeva L.A., Kamenskii A.A., Myasoedov N.F. (2012). The Nootropic and Analgesic Effects of Semax Given via Different Routes. Neurosci. Behav. Physiol..

[41] D’Adamo P., Wolfer D.P., Kopp C., Tobler I., Toniolo D., Lipp H.-P. (2004). Mice deficient for the synaptic vesicle protein Rab3a show impaired spatial reversal learning and increased explorative activity but none of the behavioral changes shown by mice deficient for the Rab3a regulator Gdi1. Eur. J. Neurosci..

[42] Cancela L., Bregonzio C., Molina V. (1995). Anxiolytic-like effect induced by chronic stress is reversed by naloxone pretreatment. Brain Res. Bull..

[43] Glazova N.Y., Atanov M.S., Pyzgareva A.V., Andreeva L.A., Manchenko D.M., Markov D.D., Inozemtseva L.S., Dolotov O.V., Levitskaya N., Kamensky A.A. (2011). Neurotropic activity of ACTH7–10PGP, an analog of an ACTH fragment. Dokl. Biol. Sci..

[44] Sazma M.A., Shields G., Yonelinas A.P. (2018). The effects of post-encoding stress and glucocorticoids on episodic memory in humans and rodents. Brain Cogn..

[45] Haider S., Naqvi F., Batool Z., Tabassum S., Sadir S., Liaquat L., Naqvi F., Zuberi N.A., Shakeel H., Perveen T. (2015). Pretreatment with curcumin attenuates anxiety while strengthens memory performance after one short stress experience in male rats. Brain Res. Bull..

[46] Padovan C., Guimarães F. (2000). Restraint-induced hypoactivity in an elevated plus-maze. Braz. J. Med. Biol. Res..

[47] Amin S.N., Hassan S.S., Khashaba A.S., Youakim M.F., Latif N.S.A., Rashed L.A., Yassa H.D. (2020). Hippocampal and Cerebellar Changes in Acute Restraint Stress and the Impact of Pretreatment with Ceftriaxone. Brain Sci..

[48] Busnardo C., Crestani C.C., Scopinho A.A., Packard B.A., Resstel L.B., Correa F.M., Herman J.P. (2018). Nitrergic neurotransmission in the paraventricular nucleus of the hypothalamus modulates autonomic, neuroendocrine and behavioral responses to acute restraint stress in rats. Prog. Neuro-Psychopharmacol. Biol. Psychiatry.

[49] Tu B.-X., Wang L.-F., Zhong X.-L., Hu Z.-L., Cao W.-Y., Cui Y.-H., Li S.-J., Zou G.-J., Liu Y., Zhou S.-F. (2019). Acute restraint stress alters food-foraging behavior in rats: Taking the easier Way while suffered. Brain Res. Bull..

[50] Hata T., Nishikawa H., Itoh E., Funakami Y. (2001). Anxiety-Like Behavior in Elevated Plus-Maze Tests in Repeatedly Cold-Stressed Mice. Jpn. J. Pharmacol..

[51] Kukharsky M.S., Ninkina N.N., An H., Telezhkin V., Wei W., De Meritens C.R., Cooper-Knock J., Nakagawa S., Hirose T., Buchman V.L. (2020). Long non-coding RNA Neat1 regulates adaptive behavioural response to stress in mice. Transl. Psychiatry.

[52] Holmes A., Parmigiani S., Ferrari P., Palanza P., Rodgers R. (2000). Behavioral profile of wild mice in the elevated plus-maze test for anxiety. Physiol. Behav..

[53] Costa-Nunes J.P., Zubareva O., Araújo-Correia M., Valença A., Schroeter C.A., Pawluski J., Vignisse J., Steinbusch H., Hermes D., Phillipines M. (2013). Altered emotionality, hippocampus-dependent performance and expression of NMDA receptor subunit mRNAs in chronically stressed mice. Stress.

[54] Zhou X., Liao W.-J., Liao J.-M., Liao P., Lu H. (2015). Ribosomal proteins: Functions beyond the ribosome. J. Mol. Cell Biol..

[55] Opmeer E.M., Kortekaas R., Aleman A. (2010). Depression and the role of genes involved in dopamine metabolism and signalling. Prog. Neurobiol..

[56] Whitton A., Treadway M.T., Pizzagalli D.A. (2015). Reward processing dysfunction in major depression, bipolar disorder and schizophrenia. Curr. Opin. Psychiatry.

[57] Ma K., Guo L., Xu A., Cui S., Wang J.-H. (2016). Molecular Mechanism for Stress-Induced Depression Assessed by Sequencing miRNA and mRNA in Medial Prefrontal Cortex. PLoS ONE.

[58] Davies S., Noor S., Carpentier E., Deviche P. (2016). Innate immunity and testosterone rapidly respond to acute stress, but is corticosterone at the helm?. J. Comp. Physiol. B.

[59] Dhabhar F.S., McEwen B.S. (1997). Acute Stress Enhances while Chronic Stress Suppresses Cell-Mediated Immunity in Vivo: A Potential Role for Leukocyte Trafficking. Brain Behav. Immun..

[60] Ray A., Gulati K., Rai N. (2017). Stress, anxiety, and immunomodulation: A pharmacological analysis. Vitam. Horm..

[61] Johnson J.D., Barnard D.F., Kulp A.C., Mehta D. (2019). Neuroendocrine Regulation of Brain Cytokines After Psychological Stress. J. Endocr. Soc..

[62] Köhler C.A., Freitas T.H., Stubbs B., Maes M., Solmi M., Veronese N., De Andrade N.Q., Morris G., Fernandes B., Brunoni A.R. (2017). Peripheral Alterations in Cytokine and Chemokine Levels After Antidepressant Drug Treatment for Major Depressive Disorder: Systematic Review and Meta-Analysis. Mol. Neurobiol..

[63] Levine J., Barak Y., Chengappa K., Rapoport A., Rebey M., Barak V. (1999). Cerebrospinal Cytokine Levels in Patients with Acute Depression. Neuropsychobiology.

[64] Gutiérrez-Meza J.M., Jarillo-Luna R.A., Rivera-Aguilar V., Miliar-García A., Campos-Rodríguez R. (2017). Cytokine profile of NALT during acute stress and its possible effect on IgA secretion. Immunol. Lett..

[65] Dygalo N.N., Bannova A.V., Sukhareva E.V., Shishkina G.T., Ayriyants K.A., Kalinina T. (2017). Effects of short-term exposure to lithium on antiapoptotic Bcl-xL protein expression in cortex and hippocampus of rats after acute stress. Biochemistry.

[66] Medvedeva E.V., Dmitrieva V.G., Povarova O.V., Limborska S.A., Skvortsova V.I., Myasoedov N.F., Dergunova L.V. (2014). The peptide semax affects the expression of genes related to the immune and vascular systems in rat brain focal ischemia: Genome-wide transcriptional analysis. BMC Genom..

[67] Stavchanskiĭ V.V., Tvorogova T.V., Botsina A.I., Skvortsova V.I., Limborskaia S.A., Miasoedov N.F., Dergunova L. (2011). V [The effect of semax and its C-end peptide PGP on expression of the neurotrophins and their receptors in the rat brain during incomplete global ischemia]. Mol. Biol..

[68] Stavchanskiǐ V.V., Tvorogova T.V., Botsina A.I., Limborskaia S.A., Skvortsova V.I., Miasoedov N.F., Dergunova L.V. (2013). The effect of semax and its C-end peptide PGP on Vegfa gene expression in the rat brain during incomplete global ischemia. Mol. Biol..

[69] Medvedeva E.V., Dmitrieva V.G., Limborska S.A., Myasoedov N.F., Dergunova L.V. (2017). Semax, an analog of ACTH(4−7), regulates expression of immune response genes during ischemic brain injury in rats. Mol. Genet. Genom..

[70] Filippenkov I., Stavchansky V.V., Denisova A.E., Yuzhakov V.V., Sevan’Kaeva L.E., Sudarkina O.Y., Dmitrieva V.G., Gubsky L.V., Myasoedov N.F., Limborska S.A. (2020). Novel Insights into the Protective Properties of ACTH(4-7)PGP (Semax) Peptide at the Transcriptome Level Following Cerebral Ischaemia–Reperfusion in Rats. Genes.

[71] Dergunova L.V., Filippenkov I.B., Stavchansky V.V., Denisova A.E., Yuzhakov V.V., Mozerov S.A., Gubsky L.V., Limborska S.A. (2018). Genome-wide transcriptome analysis using RNA-Seq reveals a large number of differentially expressed genes in a transient MCAO rat model. BMC Genom..

[72] Vyunova T.V., Andreeva L., Shevchenko K., Myasoedov N. (2018). Peptide-based Anxiolytics: The Molecular Aspects of Heptapeptide Selank Biological Activity. Protein Pept. Lett..

[73] Lee S., Hay D., Pioszak A.A. (2016). Calcitonin and Amylin Receptor Peptide Interaction Mechanisms. J. Biol. Chem..

[74] Dunlavey C.J. (2018). Introduction to the Hypothalamic-Pituitary-Adrenal Axis: Healthy and Dysregulated Stress Responses, Developmental Stress and Neurodegeneration. J. Undergrad. Neurosci. Educ..

[75] Ciccocioppo R., de Guglielmo G., Hansson A.C., Ubaldi M., Kallupi M., Cruz M.T., Oleata C.S., Heilig M., Roberto M. (2014). Restraint Stress Alters Nociceptin/Orphanin FQ and CRF Systems in the Rat Central Amygdala: Significance for Anxiety-Like Behaviors. J. Neurosci..

[76] Spitzer M., Wildenhain J., Rappsilber J., Tyers M. (2014). BoxPlotR: A web tool for generation of box plots. Nat. Methods.

[77] Chomczynski P. (1987). Single-Step Method of RNA Isolation by Acid Guanidinium Thiocyanate–Phenol–Chloroform Extraction. Anal. Biochem..

[78] Bustin S.A., Benes V., Garson J., Hellemans J., Huggett J., Kubista M., Mueller R., Nolan T., Pfaffl M., Shipley G.L. (2009). The MIQE Guidelines: Minimum Information for Publication of Quantitative Real-Time PCR Experiments. Clin. Chem..

[79] Pfaffl M.W. (2002). Relative expression software tool (REST(C)) for group-wise comparison and statistical analysis of relative expression results in real-time PCR. Nucleic Acids Res..

[80] Pfaffl M.W., Tichopad A., Prgomet C., Neuvians T.P. (2004). Determination of stable housekeeping genes, differentially regulated target genes and sample integrity: BestKeeper—Excel-based tool using pair-wise correlations. Biotechnol. Lett..

[81] Huang D.W., Sherman B.T., Lempicki R. (2008). Systematic and integrative analysis of large gene lists using DAVID bioinformatics resources. Nat. Protoc..

[82] Subramanian A., Tamayo P., Mootha V.K., Mukherjee S., Ebert B.L., Gillette M.A., Paulovich A., Pomeroy S.L., Golub T.R., Lander E.S. (2005). Gene set enrichment analysis: A knowledge-based approach for interpreting genome-wide expression profiles. Proc. Natl. Acad. Sci. USA.

[83] Babicki S., Arndt D., Marcu A., Liang Y., Grant J.R., Maciejewski A., Wishart D.S. (2016). Heatmapper: Web-enabled heat mapping for all. Nucleic Acids Res..

[84] National Center for Biotechnology Information. https://www.ncbi.nlm.nih.gov/sra/PRJNA633551.

