# Supplementary material for: Antistress Action of Melanocortin Derivatives Associated with Correction of Gene Expression Patterns in the Hippocampus of Male Rats Following Acute Stress
Source: Int J Mol Sci. 2021 Sep 17;22(18):10054. doi: 10.3390/ijms221810054 (PMC8469576; doi:10.3390/ijms221810054)
Supplement: Supplementary file 1 [file ijms-22-10054-s001.zip › Supplementary Figure S1.pptx]

## Slide 1
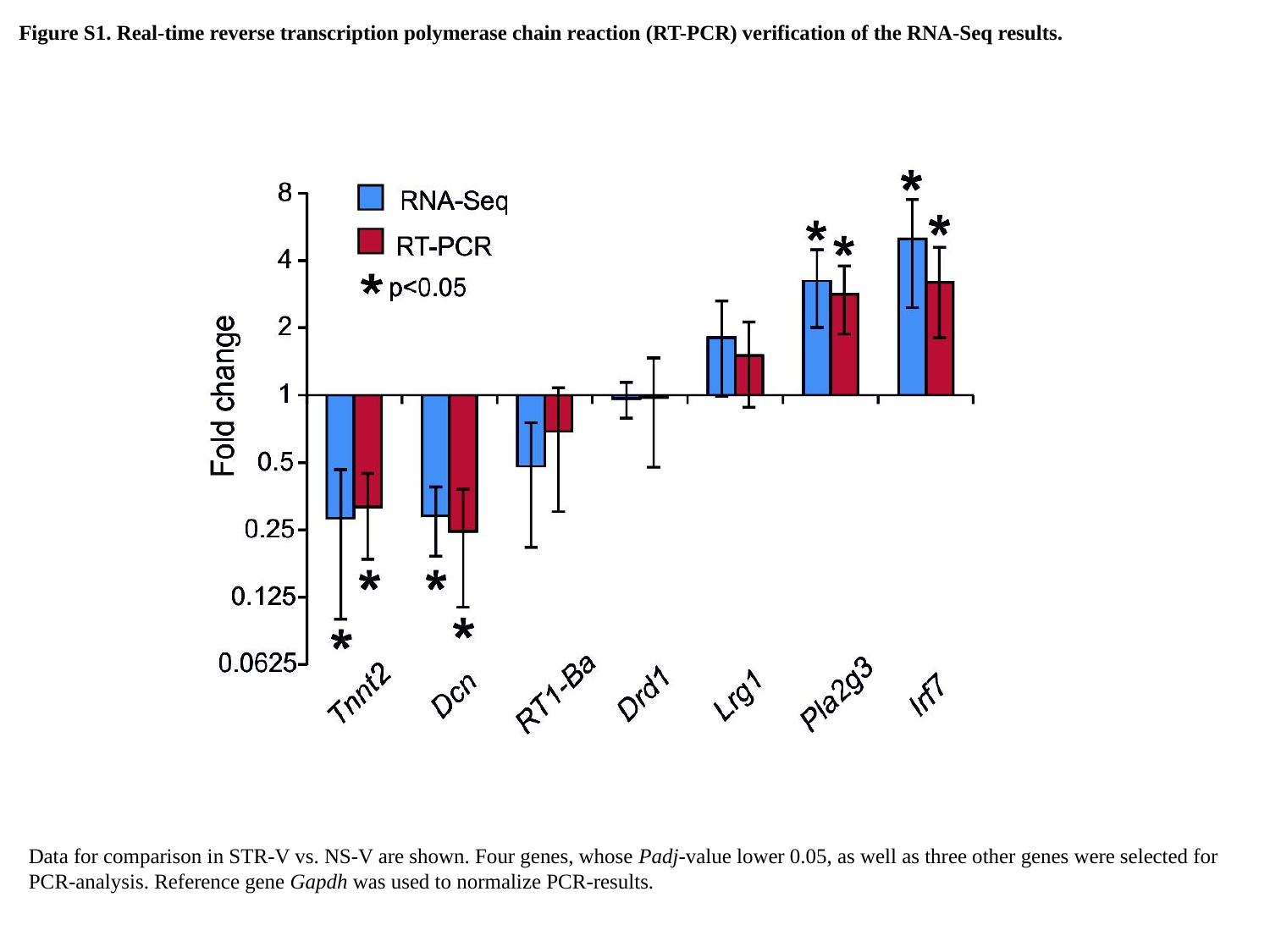

Figure S1. Real-time reverse transcription polymerase chain reaction (RT-PCR) verification of the RNA-Seq results.
Data for comparison in STR-V vs. NS-V are shown. Four genes, whose Padj-value lower 0.05, as well as three other genes were selected for PCR-analysis. Reference gene Gapdh was used to normalize PCR-results.
