# Supplementary material for: Antistress Action of Melanocortin Derivatives Associated with Correction of Gene Expression Patterns in the Hippocampus of Male Rats Following Acute Stress
Source: Int J Mol Sci. 2021 Sep 17;22(18):10054. doi: 10.3390/ijms221810054 (PMC8469576; doi:10.3390/ijms221810054)
Supplement: Supplementary file 1 [file ijms-22-10054-s001.zip › Supplementary Figure S2.pptx]

## Slide 1
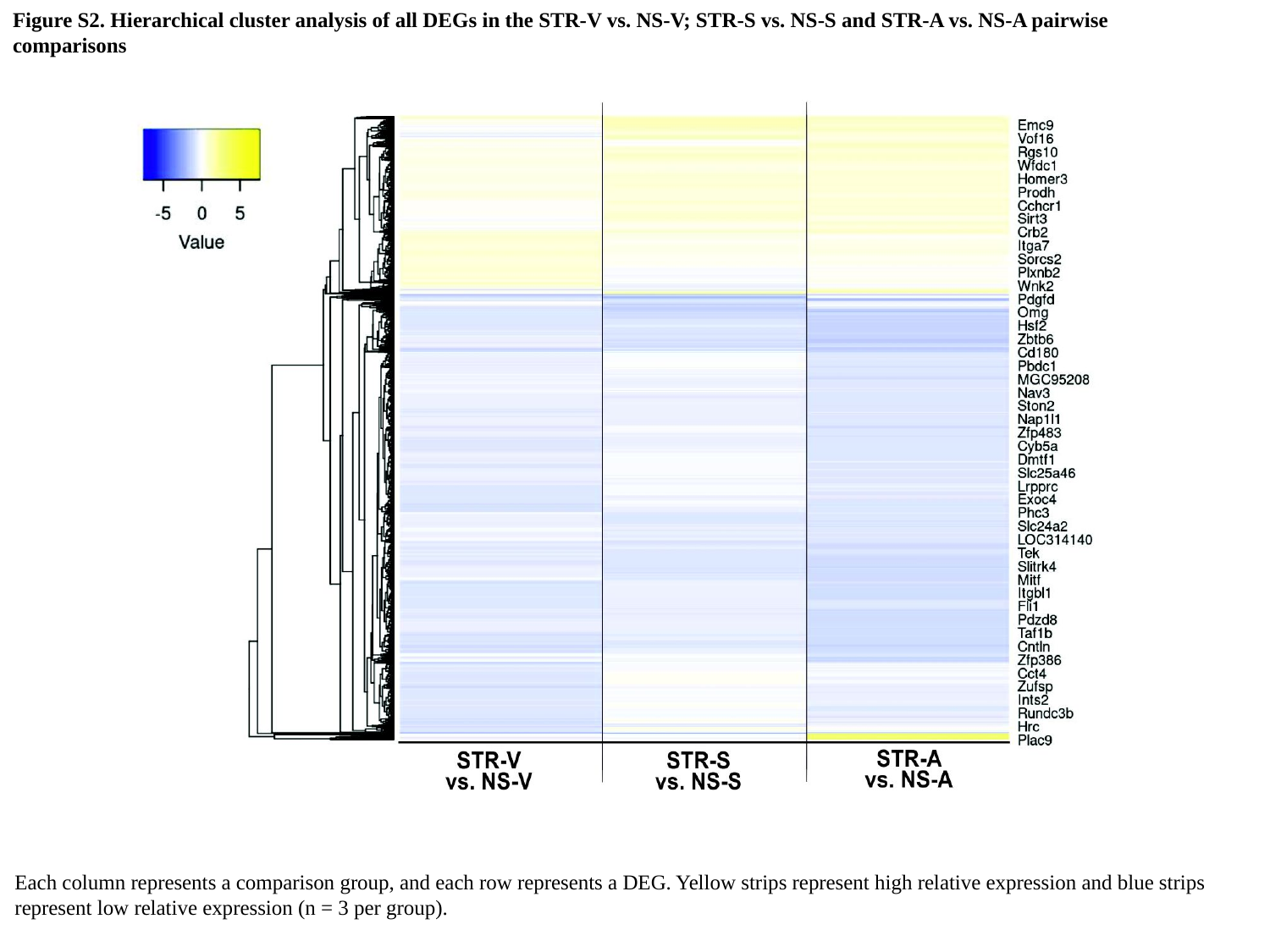

Figure S2. Hierarchical cluster analysis of all DEGs in the STR-V vs. NS-V; STR-S vs. NS-S and STR-A vs. NS-A pairwise comparisons
Each column represents a comparison group, and each row represents a DEG. Yellow strips represent high relative expression and blue strips represent low relative expression (n = 3 per group).
