# Supplementary material for: Antistress Action of Melanocortin Derivatives Associated with Correction of Gene Expression Patterns in the Hippocampus of Male Rats Following Acute Stress
Source: Int J Mol Sci. 2021 Sep 17;22(18):10054. doi: 10.3390/ijms221810054 (PMC8469576; doi:10.3390/ijms221810054)
Supplement: Supplementary file 1 [file ijms-22-10054-s001.zip › Supplementary Figure S3.pptx]

## Slide 1
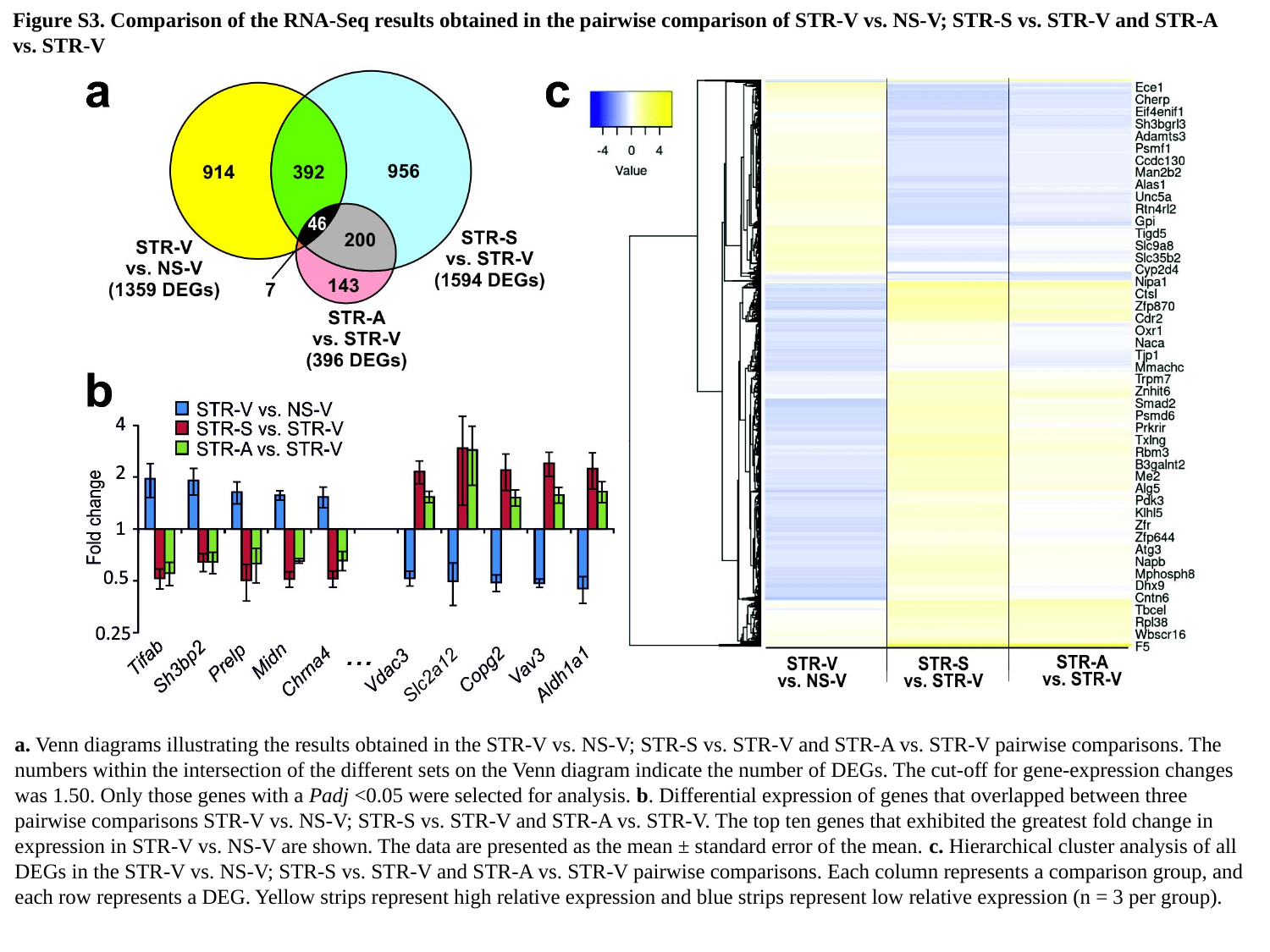

Figure S3. Comparison of the RNA-Seq results obtained in the pairwise comparison of STR-V vs. NS-V; STR-S vs. STR-V and STR-A vs. STR-V
a. Venn diagrams illustrating the results obtained in the STR-V vs. NS-V; STR-S vs. STR-V and STR-A vs. STR-V pairwise comparisons. The numbers within the intersection of the different sets on the Venn diagram indicate the number of DEGs. The cut-off for gene-expression changes was 1.50. Only those genes with a Padj <0.05 were selected for analysis. b. Differential expression of genes that overlapped between three pairwise comparisons STR-V vs. NS-V; STR-S vs. STR-V and STR-A vs. STR-V. The top ten genes that exhibited the greatest fold change in expression in STR-V vs. NS-V are shown. The data are presented as the mean ± standard error of the mean. c. Hierarchical cluster analysis of all DEGs in the STR-V vs. NS-V; STR-S vs. STR-V and STR-A vs. STR-V pairwise comparisons. Each column represents a comparison group, and each row represents a DEG. Yellow strips represent high relative expression and blue strips represent low relative expression (n = 3 per group).
